# An overview of downhill esophageal varices: a challenge for medical practice

**DOI:** 10.1080/07853890.2025.2462452

**Published:** 2025-02-04

**Authors:** Donghong Wang, Zhibin Ma

**Affiliations:** ^a^ Department of Internal Medicine, Harbin Medical University, Harbin, Heilongjiang, China; ^b^ Department of Gastroenterology and Hepatology, The First Affiliated Hospital of Harbin Medical University, Harbin, Heilongjiang, China

**Keywords:** Downhill esophageal varices, hemodialysis, mediastinal malignancy

## Abstract

**Objectives:**

Unlike the commonly seen uphill esophageal varices in clinical practice, downhill esophageal varices are caused by obstruction of the superior vena cava and azygous venous system. The predominant causes of downhill esophageal varices are hemodialysis in end-stage renal disease patients and mediastinal malignancies. The cornerstone of the treatment for downhill esophageal varices is to address the underlying primary causes. Without this, patients may suffer from recurrent bleeding, and the bleeding can be fatal.

**Methods:**

This review is primarily summarized through previous case reports. Meanwhile, it emphasizes the significance of case reports.

**Results:**

Clinicians should be conscious that esophageal varices are not necessarily caused by liver cirrhosis or non-cirrhotic portal hypertension.

**Conclusions:**

Specifically, when varices are only observed in the upper and middle esophagus, and the patient presents with evidence of superior vena cava obstruction, clinicians should be particularly vigilant for downhill esophageal varices. Moreover, a thorough investigation and definitive treatment of the underlying primary causes should be implemented.

## Introduction

Esophageal varices can be categorized into uphill and downhill types based on the direction of blood flow. Downhill esophageal varices, which are the topic of this review, are termed as such, because the blood flows from the oral side towards the cardiac side. The incidence of their detection during gastrointestinal endoscopic examinations is 0.5% [1]. Clinically, downhill esophageal varices are less prevalent than uphill ones resulting from cirrhotic and non-cirrhotic portal hypertension [2], however, due to their complex and varied causes, downhill esophageal varices should be taken seriously and emphasized. Downhill esophageal varices are easily missed before they rupture and bleed due to the lack of typical symptoms. Also, patients are frequently misdiagnosed with uphill esophageal varices secondary to liver diseases or portal hypertension [3]. These could potentially lead to incorrect treatment plans and negative consequences for patients. Downhill esophageal varices should be regarded as a red flag for potential mediastinal tumors, which is a major cause of it [4]. Once the esophageal varices rupture and hemorrhage, they may produce symptoms like hematemesis, melena, and anemia, the bleeding can also be fatal [5]. Endoscopic therapies are effective for temporizing active variceal hemorrhage, however, resolution of the underlying causes ought to be achieved as soon as possible [6]. Otherwise, the patients may suffer from recurrence of bleeding [7]. This overview provides a comprehensive review of downhill esophageal varices from various perspectives, aiming to increase clinicians’ vigilance for this medical condition.

## Pathogenesis

The venous blood originating from the esophagus initially drains into the submucosal plexus. Subsequently, this submucosal plexus channels the blood into the peri-esophageal venous plexus. From the latter plexus, esophageal veins emerge in a segmental pattern, closely following the distribution of the arterial supply. Eventually, the upper two-thirds of the submucosal plexus empties into the superior vena cava, and the lower third drains into the portal vein through left gastric vein [8].

**Figure 1.**
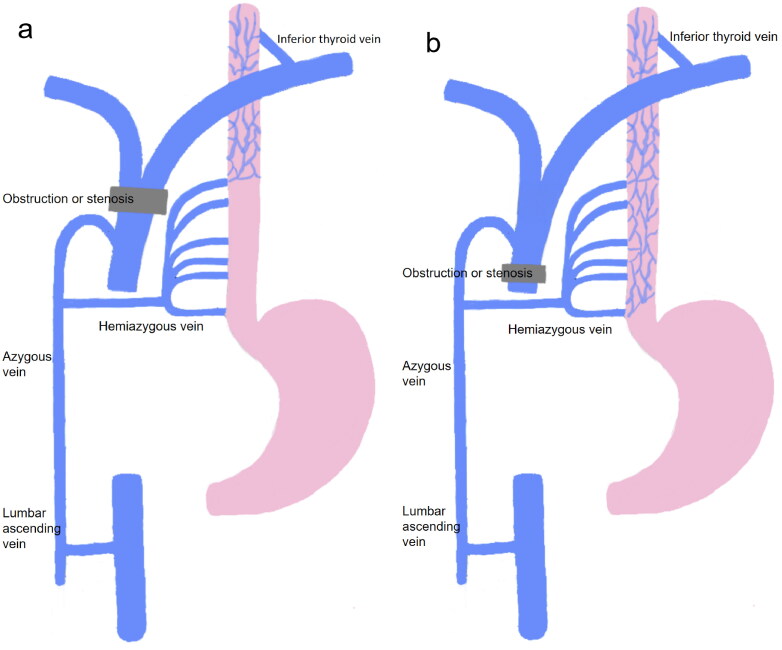
The mechanism of downhill esophageal varices. (a) Obstruction at the level of the superior vena cava. (b) Obstruction involves the superior vena cava and azygous vein system.

The elevated pressure resulting from liver diseases and portal hypertension triggers a countercurrent phenomenon between esophageal veins and the portal vein, thus resulting in the development of uphill esophageal varices [9]. Unlike uphill esophageal varices, downhill esophageal varices are not associated with liver diseases or portal hypertension. Instead, they are caused by obstruction of the superior vena cava and azygous venous system. The obstruction may be caused by internal blockage like thrombosis, and may also result from stenosis due to external compression [10,11]. When the superior vena cava is obstructed, its pressure rises significantly. The increased pressure disrupts the normal pressure gradient that exists between the esophageal veins and the superior vena cava, resulting in a reversal of blood flow direction. Initially, blood flows from the esophageal veins into the superior vena cava and the azygos venous system. However, due to the obstruction, the flow pattern is reversed, with blood now flowing from the superior vena cava back to the esophageal veins. To counteract the elevated pressure in the superior vena cava, the body activates a compensatory mechanism, which involves the establishment of collateral circulation. The esophageal venous plexus, being a collateral route, experiences an increase in blood flow. This augmented blood flow causes the veins to dilate and become tortuous, thus forming the varices. When the obstruction is only at the level of the superior vena cava, blood communicates with the esophageal veins through the inferior thyroid vein and mediastinal collateral circulation, and then returns to the heart through the azygous venous system. In this case, the varices are limited to the upper part of the esophagus, mostly in the upper third of the esophagus (Figure 1a). If the obstruction involves not only the superior vena cava but also the azygous venous system, the blood is forced to return through the inferior vena cava, and varices appear throughout the esophagus (Figure 1b) [12]. The bleeding risk of downhill esophageal varices is lower than that of uphill esophageal varices caused by portal hypertension [13]. A possible reason is that downhill esophageal varices are located deeper and have less contact with gastric acid [14]. Coagulation disorders secondary to the primary diseases of uphill esophageal varices, like liver cirrhosis, can also increase the bleeding risk [15]. Moreover, the lower pressure of the superior vena cava compared to that of the portal vein may also contribute to the lower bleeding risk of downhill esophageal varices [16].

## Etiology

### Hemodialysis for end-stage renal disease

The incidence of central venous stenosis and obstruction resulting from dialysis catheters is as high as 30% [3]. Placement of central venous catheters during dialysis results in intravascular inflammatory reactions and endothelial damage, which leads to thrombosis. Intimal hyperplasia due to blood turbulence resulting from the dialysis catheter as a foreign body in the blood vessel also contributes to superior vena cava obstruction [17]. Complication correlated with venous catheters during hemodialysis is the most common cause of bleeding from downhill esophageal varices, accounting for 27% [6]. The use of anticoagulants during hemodialysis and the coagulation disorders in patients with ESRD also increase the bleeding risk of esophageal varices [18,19].

### Mediastinal malignancy

Superior vena cava syndrome secondary to malignant tumors in the mediastinal region, like bronchial cancer, lung cancer, and lymphoma, is the most common etiology of downhill esophageal varices [20]. However, bleeding from downhill esophageal varices attributed to this etiology accounts for only 14% [6]. The mechanism includes the direct compression of the thin-walled and relatively low-pressure superior vena cava from the tumor itself or enlarged lymph nodes [21,22]. The venous thrombosis that often occurs in patients with cancer, can also lead to superior vena cava obstruction [23]. Some anticancer drugs like bevacizumab, which is used for cancer treatment, increase the risk of venous thrombosis [24,25].

### Other relatively uncommon etiologies

Other relatively uncommon cases, like goiter, Behcet’s disease, and pacemaker implantation, have been reported. A giant goiter can lead to the formation of downhill esophageal varices owing to the high volume of return blood flow. In addition, a goiter may compress the superior vena cava directly if it is retrosternal [1,11]. As for patients with Behcet’s disease, the superior vena cava obstruction is often attributed to thrombosis [26]. After pacemaker implantation, upper-extremity deep vein thrombosis may occur and develop symptoms [27]. Pacemaker leads can also induce SVC occlusion and result in downhill esophageal varices [28].

## Clinical practice

### Manifestations of the primary diseases

Patients with ESRD can present with edema in body parts like the eyelids and ankles [29]. Patients with mediastinal malignancies tend to have symptoms like chest tightness, chest pain, shortness of breath, and cough [30]. Oral ulcers, genital ulcers, and uveitis are usually observed in patients with Behcet’s [31].

### Manifestations of esophageal varices

Lots of patients are first diagnosed with bleeding from esophageal varices and present with symptoms like hematemesis, melena, and anemia. Since the bleeding risk of downhill esophageal varices is lower than that of uphill esophageal varices [32], some potential patients may experience missed diagnoses due to a lack of typical symptoms. Therefore, the actual incidence of downhill esophageal varices should be higher than the statistical incidence.

### Manifestations of superior vena cava syndrome

The most common clinical symptoms include but not limited to facial and neck edema, ectatic chest veins, headache, and watering eyes [33]. In some cases, typical SVC symptoms can be observed, even as an initial symptom [34]. However, it is noteworthy that not all patients present with the typical symptoms of SVC syndrome [35,36]. Clinicians should never exclude the diagnosis of downhill esophageal varices just because of the absence of typical manifestations of SVC syndrome.

### Diagnosis

When it comes to the diagnosis of downhill esophageal varices, it should be differentiated from uphill esophageal varices caused by portal hypertension in liver cirrhosis. The endoscopic appearance is a crucial indication for clinicians, especially when varices are only observed in the upper and middle esophagus. If a patient presents with this endoscopic appearance and has no history of liver diseases or excessive alcohol consumption, there should be a strong suspicion and thorough investigation of alternative causes of downhill esophageal varices [11]. Contrast-enhanced CT is widely used to diagnose SVC syndrome. It provides optimal visualization of the SVC, displays the range and extent of intrinsic blockage, differentiates thrombosis from external compression, and identifies collateral vessels [37]. CT angiography and magnetic resonance venography also assist in the visualization of the veins [38].

### Management

For downhill esophageal varices, the cornerstone of treatment is resolving the underlying primary cause. Some downhill esophageal varices can be relieved or even disappear after eliminating the primary cause [39]. When it comes to bleeding downhill esophageal varices, the treatments are similar to those for bleeding uphill esophageal varices, like variceal band ligation and sclerotherapy [40]. Hemostasis can often be achieved with endoscopic intervention, however, the bleeding will recur due to the underlying primary etiology not being resolved [3,7]. When injected at the level of the middle and upper esophagus, the sclerosant is likely to flow in reverse to spinal veins, thus, there is a potential risk of spinal cord infarction [14]. Also, since there has been a case of fatal pulmonary embolism due to sclerosing with polidocanol for the treatment of downhill esophageal varices, some scholars believe that ligation is safer and more reliable than sclerotherapy [41]. However, in some cases, the performing of banding is hampered by the proximity of the bleeding point to the upper esophageal sphincter [42]. As for downhill esophageal varices secondary to hemodialysis, endoscopic therapies show limited effects, whereas recanalization and angioplasty of the SVC are better solutions [43]. Patients with downhill esophageal varices secondary to giant goiter benefit from repeated segmental embolization of the thyroid arteries supplying the goiter [11].

## Discussion

Downhill esophageal varices are very likely to be missed and misdiagnosed due to the relatively lower prevalence compared to uphill esophageal varices, and their complex and varied etiologies. In order to avoid the delay of the patient’s condition and prevent recurrent bleeding, the accurate identify and definitive treatment of the underlying primary causes are urgently required [3,7]. To date, there have been no expert guidelines or consensus on the diagnosis and management of downhill esophageal varices [14]. This review is primarily summarized through reported clinical cases, and has certain limitations. Although case reports lack the evidence-based rigor of clinical trials, they remain vital resources that provide novel ideas and inspire critical thinking, which have led to some of the most remarkable discoveries in the medical domain. However, case reports have fallen out of favor in recent years due to authorship abuse and their marginal effects on the impact factor of a journal. Clinicians should still pay sufficient attention to case reports, as they serve as first-line evidence, and provide unknown and unexpected findings [44].

As a primary etiology of bleeding esophageal varices, active imaging and endoscopic surveillance should be applied to patients undergoing dialysis for the early detection of SVC obstruction and downhill esophageal varices. The specific impact of bleeding downhill esophageal varices on the prognosis of patients on ESRD dialysis needs further research. Whether anticoagulants can be used for thrombosis in the SCV and whether the use of anticoagulants will increase the risk of bleeding from downhill esophageal varices warrant further investigation. As for active variceal hemorrhage, there remains a debate over which therapy, band ligation or sclerotherapy, is more efficacious and appropriate [14]. Whether endoscopic intervention is necessary for non-bleeding varices detected during upper gastrointestinal endoscopy, after addressing the primary underlying cause, is also a topic worthy of discussion. In clinical practice, many patients tend to refuse to undergo further examination of the underlying cause of downhill esophageal varices after achieving hemostasis. This may result in rare etiologies remaining undiscovered. Commonly used hemostatic medications such as octreotide and proton pump inhibitors, are considered to have little efficacy in managing the active bleeding of downhill esophageal varices [6]. This reflects that the pharmacological treatment of downhill esophageal varices is also a necessary research orientation. Some of the downhill esophageal varices can disappear after addressing the primary cause [11,26]. However, in the previous case reports, neither the appropriate follow up interval for those varices that do not regress after treating the primary disease nor whether endoscopic sequential therapy is necessary have been mentioned. Thus, these issues require profound discussion. At present, there are insufficient studies on downhill esophageal varices, and a lack of authoritative guidelines or consensus to guide clinical diagnosis and management. The incidence of downhill esophageal varices has not been scientifically calculated, and the basic classification and bleeding risk stratification need to be improved. These issues are significant challenges for endoscopists and gastroenterologists. Multilevel, multidimensional and comprehensive guidelines and consensus are imperative. In medical practice, it’s remarkable that esophageal varices are not necessarily secondary to cirrhotic and non-cirrhotic portal hypertension. Multidisciplinary concerted work is of great importance. Clinicians should be especially vigilant when varices are only seen in the upper and middle esophagus, with no varices present in the lower segment, accompanied by the sign of SVC obstruction. This review aims to help clinicians be familiar with the clinical characteristics of various etiologies of downhill esophageal varices and improve diagnostic accuracy.

## Data Availability

No data were generated in this work.
